# Social Determinants in Self-Protective Behavior Related to COVID-19: Association Rule–Mining Study

**DOI:** 10.2196/34020

**Published:** 2022-06-15

**Authors:** Gabriel Urbanin, Wagner Meira, Alexandre Serpa, Danielle de Souza Costa, Leonardo Baldaçara, Ana Paula da Silva, Rafaela Guatimosim, Anísio Mendes Lacerda, Eduardo Araújo Oliveira, Andre Braule, Marco Aurélio Romano-Silva, Antônio Geraldo da Silva, Leandro Malloy-Diniz, Gisele Pappa, Débora Marques Miranda

**Affiliations:** 1 Departamento de Ciência da Computação Universidade Federal de Minas Gerais Belo Horizonte Brazil; 2 Instituto de Saúde Mental Baseada em Evidências Universidade Federal de Minas Gerais Belo Horizonte Brazil; 3 Associação Brasileira de Psiquiatria Brasilia Brazil; 4 Departamento de Pediatria Universidade Federal de Minas Gerais Belo Horizonte Brazil; 5 Centro de Tecnologia em Medicina Molecular Universidade Federal de Minas Gerais Belo Horizonte Brazil; 6 Faculdade de Medicina da Universidade do Porto Porto Portugal

**Keywords:** social determinants, data mining, self-protective behavior, COVID-19, protection, behavior, sanitation, characteristic, Brazil, compliance, public health, policy, mask, risk

## Abstract

**Background:**

Human behavior is crucial in health outcomes. Particularly, individual behavior is a determinant of the success of measures to overcome critical conditions, such as a pandemic. In addition to intrinsic public health challenges associated with COVID-19, in many countries, some individuals decided not to get vaccinated, streets were crowded, parties were happening, and businesses struggling to survive were partially open, despite lockdown or stay-at-home instructions. These behaviors contrast with the instructions for potential benefits associated with social distancing, use of masks, and vaccination to manage collective and individual risks.

**Objective:**

Considering that human behavior is a result of individuals' social and economic conditions, we investigated the social and working characteristics associated with reports of appropriate protective behavior in Brazil.

**Methods:**

We analyzed data from a large web survey of individuals reporting their behavior during the pandemic. We selected 3 common self-care measures: use of protective masks, distancing by at least 1 m when out of the house, and handwashing or use of alcohol, combined with assessment of the social context of respondents. We measured the frequency of the use of these self-protective measures. Using a frequent pattern–mining perspective, we generated association rules from a set of answers to questions that co-occur with at least a given frequency, identifying the pattern of characteristics of the groups divided according to protective behavior reports.

**Results:**

The rationale was to identify a pool of working and social characteristics that might have better adhesion to behaviors and self-care measures, showing these are more socially determined than previously thought. We identified common patterns of socioeconomic and working determinants of compliance with protective self-care measures. Data mining showed that social determinants might be important to shape behavior in different stages of the pandemic.

**Conclusions:**

Identification of context determinants might be helpful to identify unexpected facilitators and constraints to fully follow public policies. The context of diseases contributes to psychological and physical health outcomes, and context understanding might change the approach to a disease. Hidden social determinants might change protective behavior, and social determinants of protective behavior related to COVID-19 are related to work and economic conditions.

**Trial Registration:**

Not applicable.

## Introduction

Collectively, individual behavior is a crucial determinant of the success of measures to overcome critical conditions, such as a pandemic. During 2020-2021, Brazil had more than 20 million confirmed cases of COVID-19 and about 600,000 COVID-19–related deaths [1]. SARS-CoV-2 circulated widely, and intensive care unit (ICU) beds available reduced quickly, causing an imminent risk of health system collapse in many Brazilian states. Due to high transmission rates and a sequence of new variants, populations were caught in a conflict between the need for social distancing and economic burden [1,2].

Social restrictions with early and mandatory quarantine were supposed to be effective and were extensively recommended to contain virus dissemination [3]. Despite lockdown, isolation, and self-care campaigns, there was conflicting behavior by some people occupying the streets due to partially open commercial activities, protests, and leisure activities. Data from mobile phones showed not more than 50% of isolation in any given moment, even in critical periods of high transmission, a lack of ICU beds, and extenuated health professional teams [4]. Commonly, there is no convergence between the severity of the pandemic and individual behavior. It is interesting to note that information does not always lead to better and rational decisions. For example, in the face of proximity to death, individuals can activate some psychological defenses, such as minimizing the threat of the virus and its impact on their life [5]. In addition, individual behavior to deal with prevention depends on many factors, such as trust in the government and its strategies [6] and perception of the leaders' style to solve moral dilemmas [7]. These perceptions affected the efficacy of public policies to prevent infection during the COVID-19 pandemic.

In developed countries, re-emerging new waves of apparently more transmissible variants, driven by refusal to vaccinate, increased the risk of emergence of new resistant strains [8]. The lack of compliance with containing measures during a pandemic is not new. In 1919, Major George A. Soper published a paper in *Science*, entitled “Lessons of the Pandemic,” regarding the Spanish flu pandemic [9]. He stated that 3 main factors stand in the way of prevention: First, public indifference, when people do not appreciate the risks they run due to a lack of comprehension of the disease; second, it does not lie in human nature for a person who has only a slight cold to shut up in rigid isolation as a means of protecting others on the bare chance that it may turn out to be a really dangerous infection; and third, the highly infectious nature of respiratory infections adds to the difficulty of their control, and the disease may be transmissible before the patient is aware that they are attacked. Despite all technological progress in the past 100 years, a health crisis still requires large-scale behavior modification, with a significant social and psychological burden on individuals and their families. It was estimated that up to 40% [10] of premature deaths were accountable to individual unhealthy lifestyle decisions and behaviors [11]. It is paramount to align individual human behavior with the recommendations by public health experts.

Social, economic, psychological, and physical environments promote different changes in population behavior across stages of life [12,13]. Some social determinants, such as socioeconomic status, might delineate the distribution of mental disorders in the population, with socially disadvantaged individuals suffering a greater impact [13]. For example, there is a 2.5 times greater risk of having depression or anxiety among youth with low socioeconomic status than among those with a higher socioeconomic status [12]. An economic disadvantage also brings conditions such as compromised immune systems, diabetes, heart disease, and chronic lung diseases, resulting in higher morbidity in individuals infected by SARS-CoV-2 [12]. Those at an economic disadvantage are more likely to be exposed to the virus, susceptible to its effects, and suffer negative outcomes.

In Italy, many factors were considered as predictors of well-being in self-reports: gender (men), age (older), socioeconomic status, occupational status (unemployed), higher coping efficacy and trust in institutions, and positive attitudes toward quarantine measures [14]. During the pandemic, working conditions might have increased the risk to both COVID-19 and the related psychological burden [15,16]. There is also evidence that having COVID-19 increased anxiety, affecting home relationship engagement and critical work, and resulted in more somatic symptoms [16]. The socioeconomic burden can affect behavior and make people less willing to adopt recommended safety measures [17]. Incentives to healthier attitudes might have potential benefits, minimizing the impacts of behavior over health or shaping them according to public policy [11]. A multilevel framework should be applied to improve strategies and hence reduce new cases, deaths, and the burden of the pandemic. Policy makers must understand the dynamics of social determinants, interplaying with individual beliefs and behaviors in order to identify putative targets and plan effective care and interventions to mitigate the effects of the pandemic.

The most common self-care and protection recommendations during the COVID-19 pandemic were the universal use of facial masks, frequent handwashing or use of alcohol, and distancing when staying out (at least 1 m from someone who doesn't live with you) [18]. The cumulative protective effect might buffer transmission rates and help to control the pandemic. Considering that individual perception and behavior might change the efficacy of public policies, and part of the population reported continuing regular prepandemic life activities, 2 questions were formulated: (1) What are the characteristics of people informing careful/self-protective behavior? (2) What are their living contexts? These questions aim to better understand how we can improve conditions and strategies toward self-care, not only for the current pandemic, but also to understand the gap between presumptive information about protective measures, health promotion campaigns, and the resulting individual and societal behavior.

## Methods

### Ethical Considerations

This study was approved by the National Commission of Ethics in Research (CONEP) on May 2, 2020 (CAAE #30823620.6.0000.5149) and complied with the Declaration of Helsinki (1989). All participants were informed that the survey would take about 25 minutes to be completed. The consent form was presented on the first page of the online form, and only participants who consented to participation were further enrolled.

### Recruitment and Participants

Participants needed to be 20 years of age or more, know how to read, and have access to the internet to enroll.

Two nonprobability samples from the general population were self-selected via a survey link promoted by the Associação Brasileira de Psiquiatria (ABP) targeting the whole country at two timepoints. Participants were also invited via posts on social media. Samples were compared in a repeated cross-sectional design. The sample from timepoint 1 (T1) was collected from May 9 to June 30, 2020. The sample from timepoint 2 (T2) was collected from November 10, 2020, to January 31, 2021. Overall, there were 10,162 participants. At T1, 7802 (69.9%) individuals gave consent to the research and filled the questionnaire, whereas 3062 (23.2%) individuals participated at T2. In addition, 702 (6.9%) individuals from both T1 and T2 identified by self-generated identification codes. Cases and deaths due to COVID-19 mostly increased in most parts of the country during the collection phase.

### Measures

#### E-survey Development and Pretesting

The online survey was developed and collected through SurveyMonkey. Researchers and other collaborators tested the usability and technical functionality of the electronic questionnaire before sending it into the field. There were 61 questions displayed on 13 pages in a fixed order. No incentives were offered for survey participation. In this study, we used 4 variables from the “precautionary measures against COVID-19” question area as consequents and 11 from “sociodemographic variables” and 13 from the “work situation and economic perception” question areas as antecedents analyzed through association rule mining.

#### Sociodemographic Characteristics

The online survey contained questions investigating the participants’ gender, age, education, civil/relationship status, ethnicity, household size, residence country region, maternal education, household monthly income, and work type/situation. Regarding work type, we investigated the categories of businessperson, full-time employee, liberal profession, public/civil service, retiree/pensioner (investigated only at T2), self-employed, and unemployed. For economic classification, we used the Brazilian Economic Classification Criteria (CCEB) [19], which is a Brazilian instrument with questions about possession of durable goods and educational level of household heads. A subject score on the CCEB varies from 0 to 46, and it is classified in 1 of 6 classes with a distinct average monthly income: A (BRL 25,554.33 [US $5366.41], 2.5% of Brazilian population), B1 (BRL 11,279.14 [US $2368.62], 4.4%), B2 (BRL 5641.64 [US $1184.74], 16.5), C1 (BRL 3085.48 [US $647.95], 21.5% of Brazilian population), C2 (BRL 1748.59 [US $367.20], 26.8%), and DE (BRL 719.80 [US $151.16], 28.3%). At the time of writing, the exchange rate was BRL 1=US $0.21. In this study, we merged classes B1 and B2 into class B and classes C1 and C2 into class C.

#### Questions Related to the COVID-19 Outbreak

Sentences related to the COVID-19 outbreak were presented in a yes/no checkbox. Participants were asked to select all options that applied to their experience in the past 14 days. We based most of the questionnaire on the same questions presented in the first study on psychological impacts of the COVID-19 pandemic in China by Wang et al [12], adding questions we found appropriate for the Brazilian context at the time (ie, April 2020). The structured questionnaire consisted of 54 sentences that covered several areas. Here, we focused on questions related to precautionary measures against COVID-19, work situation, and economic perception. The questionnaire’s sentences are presented in Multimedia Appendix 1.

### Statistical Analysis: Theory/Calculation

Sociodemographic characteristics and responses on the COVID-19 questionnaire were described. Venn diagrams were used to visually describe the frequency of participants adopting at least 1 of the following preventive actions: (1) stay at least 1 m apart from people when out of the house; (2) sanitize hands with alcohol gel (70% ethyl alcohol) or wash hands for at least 20 seconds, whenever possible, when out of the house; and (3) only leave home when extremely necessary and wearing a face covering. We also depicted the frequency of participants who “kept going outdoors (leaving home) for work as usual.” One diagram was made for each timepoint investigated.

### Association Rule Mining

Research questions were answered by formulating our problem as a frequent pattern–mining task [20]. A pattern is a set of question-answer pairs, where the possible answers that compose the pair are specific to each question. A pattern is frequent when a number of subjects present a given pattern in their responses and the number is above a threshold. These frequent patterns can be used to generate association rules. An association rule follows an if-then format and is used to express how often 2 or more answers to questions of interest are associated with each other in the database. For example, we may find an association rule that says that *if* a subject is of the female gender, *then* it is frequently associated with COVID-19-protective behaviors, such as “only leaves home when extremely necessary and wearing a face covering” and “stays at least 1 m apart from people when out of the house.”

In short, association rules are generated from a set of answers to questions, also called items, that co-occur with a given frequency. Both the rule antecedent (the *if* part of the rule) and the rule consequent (the *then* part of the rule) may be formed by the answers to more than 1 question, but the set of answers that compose the rule has to occur together with the same frequency. The association between a set of answers to different questions is usually measured using 3 traditional metrics of interestingness: support, confidence, and lift.

Support shows how popular a set of question-answer pairs is, and is measured using the proportion of subjects who answered according to that set. For example, if we have responses from 100 subjects in the database and 70 (70%) of them only leave the house with a mask, the support of the answer “only leaves home when extremely necessary and wearing a face covering” is equal to 70/100 = 0.7. Confidence, in turn, measures how likely it is that a person gives a set of question-answers Y, given they gave a set of question-answers X, that is, the conditional probability of Y given X. Confidence is measured considering the frequency (support) of X and Y appearing together over the frequency (support) of X alone. One problem with confidence is that it may not capture the importance of the association, as it just accounts for the popularity of 1 question in the denominator.

The third popular metric, lift, solves this major drawback of confidence by quantifying to what extent the observed joint probability of X and Y deviates from the expected joint probability of them; in practice, it is the ratio between these 2 joint probabilities. A lift value of 1 means no correlation exists between X and Y, that is, the observed co-occurrence comes from the margins. A value greater than 1 means X and Y are positively correlated, and a value smaller than 1 means X and Y are negatively correlated. Replacing X and Y by the answers to questions from the pool, we were able to identify answers associated with both sociodemographic, COVID-19–related work situation, and economic perceptions, and adoption of human protection measures to prevent COVID-19 contamination and spread.

In this analysis, we used the Apriori algorithm [21] to determine the association rules. The support was a user-defined parameter. We used a minimum support of 5%, which establishes the minimum frequency of any question-answer pair to be considered relevant for the sake of an association rule, and a minimum confidence of 68%. For more details on frequent-pattern mining, please refer to Multimedia Appendix 1.

After the rules were generated a priori, we selected those that had in their consequent answers to questions related to measures individually taken to suppress COVID-19 transmission and contamination. We divided these rules into 2 groups: (1) those describing people who continued with their habits and lifestyle regardless of the pandemic and (2) those who were adopting at least 1 of the protection recommendations. The first group reported to continue going out normally regardless of the pandemic (“kept moving outdoors [leaving home] for work as usual”). The second group involved people who reported to take at least 1 of the following protective measures: (1) stay at least 1 m apart from people when out of the house; (2) sanitize hands with alcohol gel (70% ethyl alcohol) or wash hands for at least 20 seconds, whenever possible, when out of the house; or (3) only leave home when extremely necessary and wearing a face covering.

## Results

### Study Sample

The study sample was composed of individuals from the Brazilian adult population who have access to the internet and a computer. It was a population with predominance of women, Whites, married people, high education, from all Brazilian regions, and mostly middle class, living in a house with 3-5 people, at both timepoints. Tables 1-3 list the participants’ sociodemographic characteristics, precautionary measures taken, and work situation and economic perceptions, respectively.

At T1, 131 (6%) individuals reported going out normally. At T2, 6 months later, 172 (31%) individuals reported going out normally. Despite the increase in people going out normally, most participants reported the use of protective measures against COVID-19.

**Table 1:** Sociodemographic characteristics during the COVID-19 pandemic at 2 cross-sectional timepoints.

Characteristics			T1^a^ (May-June 2020; N=7802), n (%)		T2^b,c^ (November 2020-January 2021; N=3062), n (%)
**Gender**					
	Female	5366 (68.8)		2180 (71.2)	
	Male	1148 (14.7)		594 (19.4)	
	Missing	1288 (16.5)		288 (9.4)	
**Age (years)**					
	18-19	98 (1.3)		70 (2.3)	
	20-29	1188 (15.2)		604 (19.7)	
	30-39	1601 (20.5)		735 (24.0)	
	40-49	1464 (18.8)		579 (18.9)	
	50-59	1133 (14.5)		429 (14.0)	
	60-69	599 (7.7)		211 (6.9)	
	70-90	118 (1.5)		41 (1.3)	
	Missing	1601 (20.5)		393 (12.9)	
**Education**					
	No schooling	21 (0.3)		2 (0.1)	
	Doctorate degree (PhD)	429 (5.5)		156 (5.1)	
	Elementary school diploma/incomplete junior high school	232 (3.0)		78 (2.5)	
	Incomplete elementary school	91 (1.2)		18 (0.6)	
	High school diploma/incomplete higher education	1871 (24.0)		783 (25.6)	
	Master's degree	638 (8.2)		312 (10.2)	
	Higher education degree	3079 (39.5)		1427 (46.6)	
	Missing	1441 (18.3)		286 (9.3)	
**Ethnicity**					
	Asian/Oriental	108 (1.4)		37 (1.2)	
	White	3128 (40.1)		1403 (45.8)	
	Indigenous	20 (0.3)		4 (0.1)	
	Brown	1173 (15.0)		551 (18.0)	
	Black	244 (3.1)		133 (4.3)	
	Missing	3129 (40.1)		934 (30.6)	
**Marital status**					
	Married/cohabitation	3206 (41.1)		1226 (40.0)	
	Divorced	801 (10.3)		257 (8.4)	
	Single	2204 (28.2)		1237 (40.4)	
	Widowed	33 (0.4)		46 (1.5)	
	Missing	1558 (20.0)		296 (9.7)	
**Work type**					
	Full-time employee	1279 (16.4)		580 (19.0)	
	Self-employed	947 (12.1)		285 (9.3)	
	Unemployed	1431 (18.3)		577 (18.8)	
	Liberal professional	735 (9.4)		326 (10.6)	
	Public/civil servant	1871 (24.0)		663 (21.7)	
	Retiree/pensioner	N/A^d^		202 (6.6)	
	Businessperson	N/A		75 (2.4)	
	Missing	1539 (19.8)		354 (11.6)	
**Economic class (BRL)**					
	A: BRL 25,554.33 (US $5366.41)^e^ average household income	889 (11.4)		970 (31.7)	
	B: BRL 11,279.14 (US $2368.62) or BRL 5641.64 (US $1184.74) average household income	2435 (31.2)		485 (15.8)	
	C: BRL 3085.48 (US $647.95) or BRL 1748.59 (US $367.20) average household income	1242 (15.9)		1165 (38.1)	
	DE: BRL 719.80 (US $151.16) average household income	125 (1.6)		442 (14.4)	
	Missing	3111 (39.9)		N/A	
**Monthly household income (BRL)**					
	≤500 (US $102.64)	105 (2.6)		11 (1.0)	
	501-1000 (US $102.85-$205.28)	235 (3.0)		51 (1.7)	
	1001-1500 (US $205.49-$307.92)	349 (4.5)		116 (3.8)	
	1501-2000 (US $308.12-$410.56)	368 (4.7)		117 (3.8)	
	2001-2500 (US $410.76-$513.20)	267 (3.4)		125 (4.1)	
	2501-3000 (US $513.40-$615.84)	382 (4.9)		166 (5.4)	
	3001-4000 (US $616.04-$821.12)	417 (5.3)		213 (7.0)	
	4001-5000 (US $821.32-$1026.40)	429 (5.5)		250 (8.2)	
	5001-10,000 (US $1026.60-$2052.80)	970 (12.4)		550 (18.0)	
	10,001-25,000 (US $2053.00-$5131.99)	765 (9.8)		385 (12.6)	
	≥25,001 (US $5132.20)	269 (3.4)		105 (3.4)	
	Missing	3246 (40.5)		962 (31.0)	
**Household size**					
	1 person	470 (6.0)		249 (8.1)	
	2 people	1286 (16.5)		626 (20.4)	
	3-5 people	2557 (32.3)		1189 (38.8)	
	6 people or more	172 (2.2)		62 (2.0)	
	Missing	3317 (42.5)		936 (30.7)	
**Maternal education**					
	No schooling/incomplete elementary school	769 (9.9)		331 (10.8)	
	Elementary school diploma/incomplete junior high school	1085 (13.9)		431 (14.1)	
	Junior high school diploma/incomplete high school	541 (6.9)		248 (8.1)	
	High school diploma/incomplete higher education	1149 (14.7)		571 (18.6)	
	Higher education degree	1099 (14.1)		541 (17.7)	
	Missing	3159 (40.5)		940 (30.7)	
**Brazilian geographic region**					
	North	273 (3.5)		51 (1.7)	
	Northeast	961 (12.3)		230 (7.5)	
	Central-west	362 (4.6)		126 (4.1)	
	Southeast	3624 (46.4)		2058 (67.2)	
	South	1076 (13.8)		313 (10.2)	
	Missing	1506 (19.4)		284 (9.3)	

^a^T1: timepoint 1.

^b^T2: timepoint 2.

^c^T1 and T2 were 6 months apart.

^d^N/A: not applicable.

^e^An exchange rate of BRL 1=US $0.21 was applied.

**Table 2:** Precautionary measures against COVID-19 in the past 14 days at 2 cross-sectional timepoints.

Characteristics		T1^a^ (May-June 2020; N=7802), n (%)	T2^b,c^ (November 2020-January 2021; N=3062), n (%)
**Stays at least 1 m apart from people when out of the house**			
	No	3277 (42)	1134 (37)
	Yes	4525 (58)	1928 (63)
**Sanitizes hands with alcohol gel (70% ethyl alcohol) or washes hands for at least 20 seconds, whenever possible, when out of the house**			
	No	2939 (38)	842 (27)
	Yes	4863 (62)	2220 (73)
**Only leaves home when extremely necessary and wearing a face covering**			
	No	2818 (36)	1279 (42)
	Yes	4984 (64)	1783 (58)
**Keeps moving outdoors (leaving home) for work as usual**			
	No	7679 (98)	2900 (95)
	Yes	123 (1.6)	162 (5.3)

^a^T1: timepoint 1.

^b^T2: timepoint 2.

^c^T1 and T2 were 6 months apart.

**Table 3:** COVID-19 pandemic work situation and economic perceptions at 2 cross-sectional timepoints.

Characteristics		T1^a^ (May-June 2020; N=7802)	T2^b,c^ (November 2020-January 2021; N=3062)
**Feels more productive at work, n (%)**			
	No	7126 (91.3)	2709 (88.5)
	Yes	676 (8.7)	353 (11.5)
**Feels less productive at work, n (%)**			
	No	5616 (72.0)	1980 (64.7)
	Yes	2186 (28.0)	1082 (35.3)
**Already worked from home before COVID-19, n (%)**			
	No	7423 (95.1)	2871 (93.8)
	Yes	379 (4.9)	191 (6.2)
**Working or studying from home (home-office), n (%)**			
	No	5110 (65.9)	1781 (58.2)
	Yes	2692 (34.5)	1281 (41.8)
**Started using video calling apps/software often, n (%)**			
	No	5144 (65.9)	1606 (52.4)
	Yes	2658 (34.1)	1456 (47.6)
**Working under reduced hours or taking turns with coworkers, n (%)**			
	No	6999 (89.7)	2819 (92.1)
	Yes	803 (10.3)	243 (7.9)
**Waiting social distancing rules' suspension to go back to working or studying, n (%)**			
	No	7213 (92.5)	2963 (96.8)
	Yes	589 (7.5)	99 (3.2)
**Need to leave home for work but is afraid of COVID-19, n (%)**			
	No	6,981 (89)	2,499 (82)
	Yes	821 (11)	563 (18)
**Afraid of not being able to deal with present or yet-to-come financial difficulties, n (%)**			
	No	5,663 (73)	2,216 (72)
	Yes	2,139 (27)	846 (28)
**Believes that economic struggles related to social distancing measures will be overcome soon (recovering will take 1 or 2 years after economic activity reopening/normalization), n (%)**			
	No	5791 (74.2)	2316 (75.6)
	Yes	2011 (25.8)	746 (24.4)
**Believes that economic struggles related to social distancing measures will last longer (recovering will take at least 2 years or more after economic activity reopening/normalization), n (%)**			
	No	4347 (55.7)	1412 (46.1)
	Yes	3455 (44.3)	1650 (53.9)
Average hours worked per day, mean (SD)		6.63 (3.75)	7.08 (3.65)

^a^T1: timepoint 1.

^b^T2: timepoint 2.

^c^T1 and T2 were 6 months apart.

### Association Rule Mining

**Figure 1:**
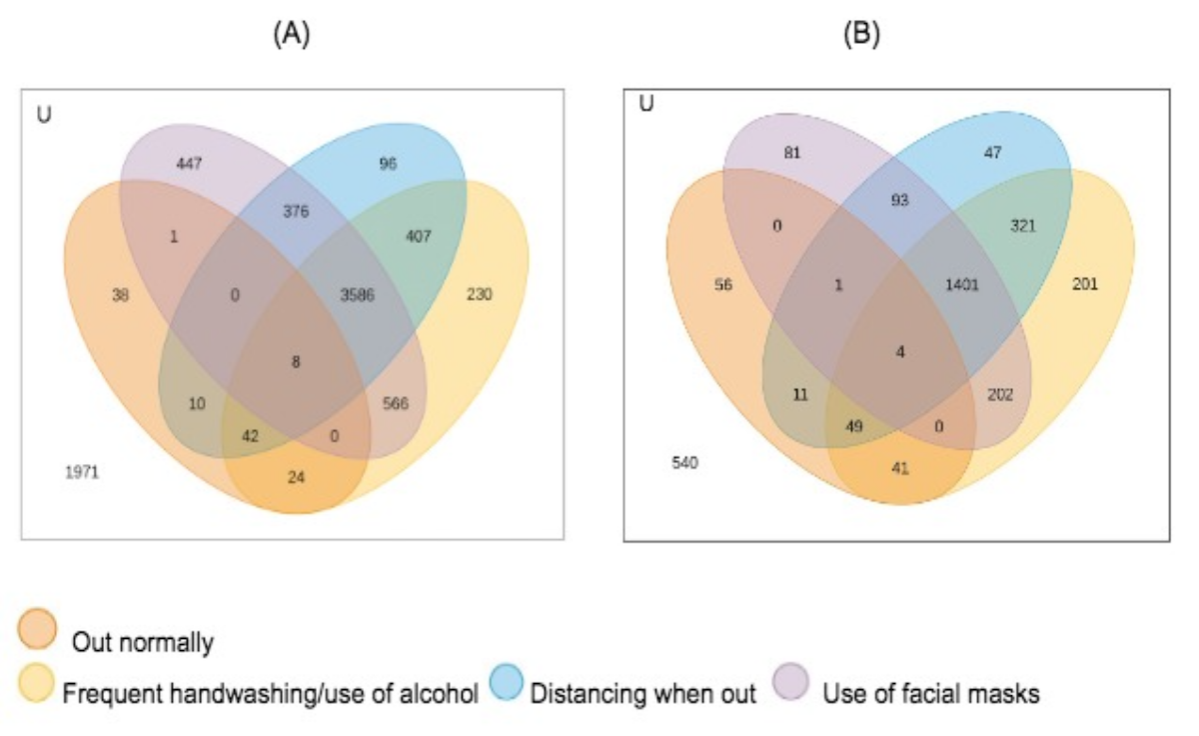
A Brazilian profile (Venn diagrams) of adoption of human protection measures to prevent COVID-19 contamination and spread: frequency of people who (1) stay at least 1 m apart from people when out of the house; (2) sanitize hands with alcohol gel (70% ethyl alcohol) or wash hands for at least 20 seconds, whenever possible, when out of the house; (3) only leave home when extremely necessary and wearing a face covering; or (4) keep moving outdoors (leaving home) for work as usual. (A) COVID-19-preventive measures’ profile at T1 (May-June 2020) (N=7802). (B) COVID-19-preventive profile at T2 (November 2020-January 2021) (N=3062). T1: timepoint 1; T2: timepoint 2.

After filtering the association rules by their consequents to obtain people who reported following at least 1 of 4 selected behaviors, we obtained a set of 1694 rules for the data collected at T1 and 2490 rules for data collected at T2. Figure 1 shows Venn diagrams of the distribution of rule consequences and the number of people who were covered by the rules, considering different protective behaviors followed during the pandemic: going out normally, frequent handwashing and use of alcohol, keeping distance when out of the house, and use of facial masks. The number of people who reported to continue going out normally represents less than 5% of the population, and hence, they did not appear in any rules, as the minimum support was set to 5%. However, we added this behavior to the diagram for completeness. Circles in a Venn diagram can overlap partially, overlap completely, or even be separate, letting one easily see the relationship between different groups of people with different sets of protective measures. From the diagram, only 45 (0.6%) and 56 (1.8%) individuals at T1 and T2, respectively, reported going out normally without taking any protective measures (frequent handwashing and use of alcohol, keeping distance when out of the house, and use of facial masks). Moreover, 9 (0.1%) and 4 (0.1%) individuals at T1 and T2, respectively, reported going out normally but taking all protective measures. Most individuals, 3711 (47.6%) at T1 and 1401 (45.8%) at T2, reported adopting all protective measures.

**Figure 2:**
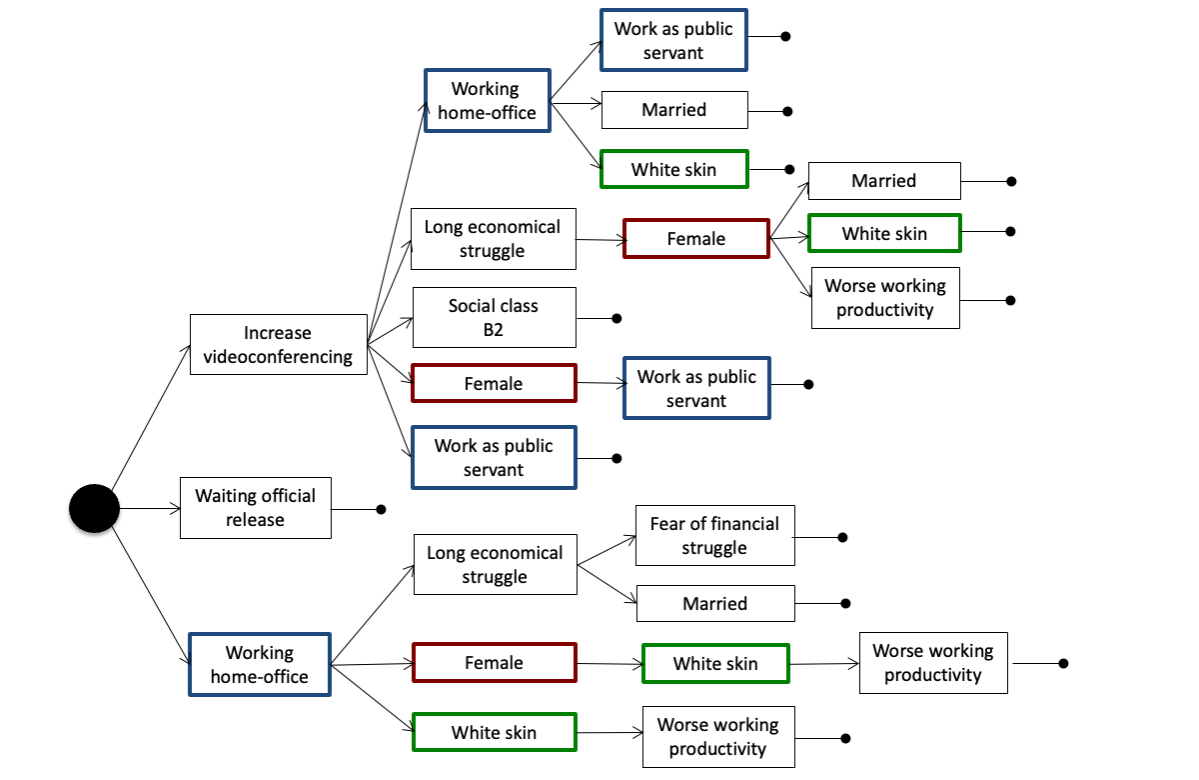
Rules generated considering the answers of people who took care of themselves in the first round of questionnaires.

**Figure 3:**
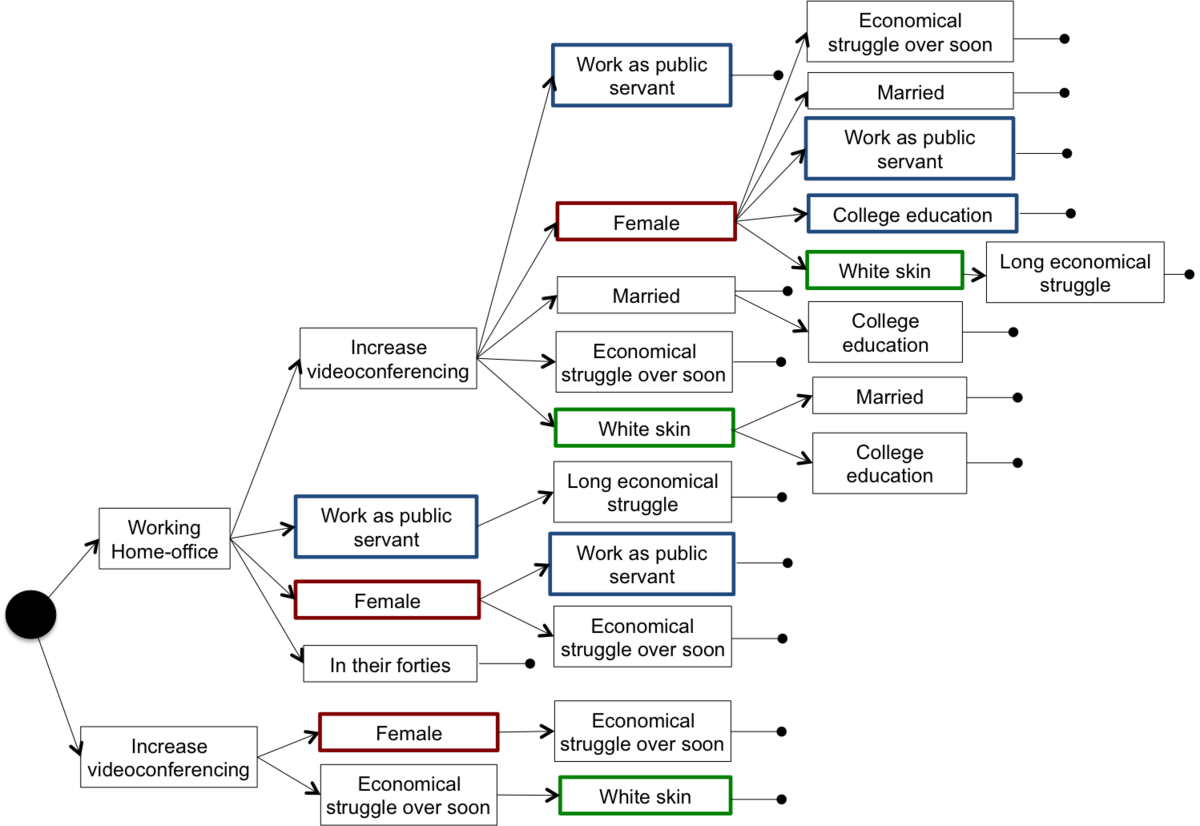
Rules generated considering the answers of people who took care of themselves in the second round of questionnaires.

Figures 2 and 3 show the set of association rules generated from the data gathered in the first and second round of surveys, respectively, when considering people that took the 3 protective measures all together. The closed circle at the end of a sequence of answers indicates the end of a rule. From a total of 32,877 rules generated, considering all possible consequents, 11 (0.03%) included all the protective measures according to participants’ answers in the first questionnaire (ie, the 3711, 47.6%, individuals in the intersection of the Venn diagram on the left in Figure 1) and 17 (0.03%) rules showed the same information for the 1401 (45.8%) participants of the second round in the shaded area representing all protective behaviors in the Venn diagram on the right in Figure 1. All rules in both figures had their confidence ranging from 0.681 to 0.736 and their lift in the (1.595,1.637) interval. These values indicated a high chance of 1 of the answers being associated with the next. Note that there were no patterns regarding the few people who were going out normally.

Reported fears included the economic struggle, fear of the disease, and fear of the potential to transmit it to their families.

## Discussion

### Principal Findings

To achieve the desired behavior for infection control (ie, extensive use of self-protective measures), increased use of videoconferencing and the possibility to work from home were present in all rules that explained better self-care behavior. The ability to change working conditions was combined with self-reports of being White, with a high educational profile and age around forties. A fear of economic struggle, in the short or the long term, composed many rules of preventive behavior. These findings might clarify hidden socioeconomic features associated with self-care measures. After 6 months, the rules were similar, and a feature related to work stability was evidenced: “being a public servant.” Public servants in Brazil have tenure, and in many positions, they are allowed to perform their activities from home. These findings suggest that social distancing and self-care protection were implemented by those who were able to follow the stay-at-home policy, unveiling potential social disparities in health care.

The COVID-19 pandemic presented some particularities useful to help understanding the dissociation between the information given and the consequences of behavior. In Brazil, we observed a dissociation between the information given by health authorities and people's reactions in terms of individual and collective care [2]. It was not only a public health problem but also, on a large scale, an information crisis. In China, data from a 3-phase survey, collected during the first wave of COVID-19, showed low cooperation with prevention and control measures in the early stages, followed by a gradual increase as the pandemic progressed [22]. We aimed to understand the population's perception of the need for self-care and social distancing, considering the observed individual behavior and its consequences. We observed, in a large mental health online data set collected from May to June 2020, that a major part (75%) of the population reported being at home, believing in the potential severity of COVID-19, and trying to keep social distancing practices. Data was collected at 2 timepoints. In both, most of the assessed population did report using at least 2 self-care measures. Interestingly, the percentage of participants who followed the protective measures was almost the same in both periods, even with the significant increase in COVID-19 cases and deaths in Brazil. Most enrolled individuals reported not believing in information provided via the internet and conventional media (television and radio). However, they reported knowing how to protect themselves against COVID-19 and adopted self-care measures, showing that the information was reaching the target.

We did not focus only on short-term thinking about the current pandemic but also focused on social determinants of self-care behavior. Individuals with unfavorable economic, social, and environmental conditions have fewer buffers and suffer stronger consequences of cumulative stress [12]. In extreme situations, such as the pandemic, the presence of a social buffer can facilitate control and determine the individual risk for developing long-term mental health disorders. In a 2-month follow up of a Mexican population, financial and security situations did not change but increased the risk for distress [23]. So, social determinants might not only be important for compliance with preventive measures and minimize new cases but also be important to avoid long-term consequences of the pandemic. Behavioral changes can be influenced, for example, by economic rewards, boosting cooperation among people, and should be considered in designing more efficient public health policies.

Financial incentives to modify behavior are cost-effective and might induce quick responses [24]. Thus, using financial incentives or other extrinsic motivations might be a strategy for governments and private organizations to improve compliance with health measures in similar conditions [25]. Infrequent behaviors, such as those required in a pandemic or in a disaster, are good targets for financial incentives, but the use of extrinsic reward is also associated with lower self-motivation, and sustained behavior seems less impacted by the incentive [11]. It might be a cost-effective strategy, especially in middle- and low-income countries, where the response depends on what people have, rather than what they can have [11]. Although extrinsic motivators might be a game changer, there is a need to better understand the strategies to sustain wished behaviors. The Brazilian government initiated many strategies during the pandemic to minimize economic burden on small and midsize businesses and vulnerable individuals [2]. The impact of these aids needs to be better known to understand the impact of financial incentives on changing behavior; however, it has been a difficult population to reach using online strategies. For a while, with this data, we only observed the importance of work's stability and related features to follow self-protective care. Further studies on evaluation of interventions with extrinsic motivators are still necessary.

Information, misinformation, fake news, and disinformation coexist in social media [26], which generates confusion, making it harder to attribute credibility to information and to educate the population on necessary health policies. In this regard, one should consider Brazil's inequality [27]. With a Gini Index of 0.849, Brazil is the fifth country in the inequality rank. Wilkinson and Pickett [28] showed that trust levels are lower in countries and states where income differences are greater. Likewise, Frank [29] gathered data from the International Social Survey Program (ISSP), with 48,651 subjects from 33 countries, and participants indicated their level of agreement with the statement “There are only a few people I can completely trust.” It was found that income inequality is correlated with country differences in trust (r=−0.51). Societies with low levels of trust may lack the ability to create the kind of social support and connections that promote health and successful aging [29]. Brazil specifically has remarkably low levels of social and interpersonal trust (5%)—in fact, 1 of the lowest in the world [30]. Since trust plays a key role in the creation of knowledge [31] and sustaining well-being outcomes, it is questionable whether the potential lack of trust among Brazilians also influences their trust in information disseminated by health organizations and had a significant impact on the spread of COVID-19 in the country.

Despite miscommunication and a lack of interpersonal trust, people reported awareness of self-protective measures [31]. Formal education affects a range of outcomes across life, such as adaptability to different standards, including switching to working from home, which was a common finding among those complying with protective measurements. It was a sample with a high educational profile, which certainly biased the responses and had an impact on the high adherence to self-care protection. In fact, people considered self-protected were those working from home and fearing the prospect of long-term impact and economic struggle.

In an unequal country, governmental financial aid to low-income families was essential to allow staying at home during the pandemic, as an act of solidarity emphasized by public health services as crucial for fighting COVID-19. Having people constantly present at work might potentially compromise contamination control. Thus, working from home seems to help mitigate the pandemic’s impacts. Based on our data, governments should consider early and enough financial aid to promote adherence to health protective measures. In contrast, long and intermittent stay-at-home measures and a lack of mental health buffers might impair the well-being and health of children, adolescents, and adults. Working from home also had an impact on both mental and physical health [32]. Factors such as lack of communication with coworkers, distractions, children at home, and adjustment of working hours are factors that influence well-being related to home-office [32]. Considering the effect of working from home on mental health, it is possible that people decided to gradually return to the workplace regardless of known risks. Incentives to work from home must be coupled with the development of strategies to improve the well-being of those at home-office and their families. A long-term and multifactorial vision of the COVID-19 pandemic will be fundamental to evaluate and understand the ramifications of the social distancing strategies adopted worldwide.

### Limitations

Some constraints must be addressed. Besides having a representative sampling of the Brazilian population, we had underrepresentation in the lowest economic classes, which was particular for the data collection strategy based on online access. In our sampling, there was a clear bias of access to the internet and to the online survey. However, as the economic and social features prevail, it is reasonable to infer that the effects might be stronger in more vulnerable populations.

### Conclusion

Stable economic conditions and the possibility of working from home sound as an organizing social strategy to promote the use of self-care measures in a pandemic. The use of self-care protective measures is determined by social determinants that should be considered by policy makers.

## Appendix

Multimedia Appendix 1 Questionnaires without copyright protection.

## Abbreviations

ICU: intensive care unit
T1: timepoint 1
T2: timepoint 2

## Data Availability

De-identified participant data and a data dictionary will be made available under reasonable request to Prof. Malloy-Diniz.
